# Drug Idolatry in Dental Medicine

**Published:** 1912-01-15

**Authors:** Hermann Prinz

**Affiliations:** Professor of Dental Materia Medica, Therapeutic and Pathology, Washington University Dental School, St. Louis, Mo.


### DRUG IDOLATRY IN DENTAL MEDICINE.

BY HERMANN PRINZ, D.D.S., M.D.,
PROFESSOR OF DENTAL MATERIA MEDICA, THERAPEUTIC AND PATHOLOGY, WASHINGTON UNIVERSITY DENTAL SCHOOL, ST. LOUIS, MO.

Within the last fifty years the theories regarding the pharmacologic action of drugs have undergone remarkable changes. Empiricism in medicine has held sway ever since remedies were used for the purpose of alleviating diseases, and it was only through the introduction of experimental pharmacology in the early sixties of the last century that a slow but radical change in the administration of drugs took place. The science of modern pharmacology is based on Virchow's conception of cellular pathology, and with its introduction into general medicine, in 1858, the humoral pathology of Hippocrates received its death-blow.

The growing tendency to overdrugging received a serious shock through the introduction (in 1810) of Hahnemann's method of treating disease with infinitesimally small doses, which, combined with other extreme changes in therapeutics, resulted in the foundation of the homeopathic school.

Up to this period no definite knowledge regarding drug action had been available to the practicing physician, and as a consequence of the empiric administration of drugs it became customary to poke fun at those who regarded them as necessary in the treatment of disease. Especially Skoda and Dietl, of the Vienna school (about 1850), expressed most eratic views in regard to drug medication, and both extremists carried the idea of drug nihilism to such an extent as to almost eliminate materia medica from the curriculum of the study of medicine.

### MODERN SYSTEMATIC CLASSIFICATION OF DRUGS.

A systematic classification of drugs according to their pharmacologic action was introduced by Buchheim in 1870, and since the appearance of "Grundriss der Arzneimittellehre," by Schmiedeberg, in the early eighties of the last century, a revolution in drug medication has taken place. This revolution was made possible only by the complete elimination of empiricism, and by utilizing the results obtained from experimental work on healthy and artificially diseased animals and, to some extent, on man. Aside from the above-named experimenters, such men as Magendie, Beaumont, Claude Bernhard, B. W. Richardson, Crum Brown, Frazer, Binz, Liebreich, Lauder Brunton, Filehne, Kobert, Ehrlich, Cushney, Abel, Pawlov, and others too numerous to mention have paved the way in the past or are still actively engaged in solving the intricate problem of drug action, and thereby have created a new branch in biological science known today as experimental therapy. Unfortunately, the dental profession has been slow in keeping pace with the progress made in general pharmacology, and as a consequence there is still much empiricism involved in the practice of dental medicine. Broadly speaking, there is no excuse for such laxity. The last decade offers ample proof of the immense effort which has been made to place dental therapeutics on a rational basis; yet many notions prevail in the minds of some practitioners regarding the action of certain remedies which are not in harmony with the modern conception of the physiologic action of drugs. The stereotyped prescriptions which are so often displayed in current dental literature, and the consequent practice of "making the disease fit the remedy," are much to blame for this pharmacologic idolatry. Even some of the text-books persist in the transmission of certain antiquated views in regard to the therapeutic action of drugs. Very recently the writer had occasion to look over many hundred questions relating to the subject of materia medica and therapeutics as asked by various state boards, and he was rather surprised at the peculiar conception of pharmaco-therapy which these questions displayed on the part of the examiners.

The graduate fresh from college, usually starts out with a long list of drugs, ready to combat all diseases. When he gets his first patient with some difficult ailment, where drug administration is indicated, he finds that the remedy which he has chosen is utterly incapable of influencing the existing conditions; his faith receives a severe shock, and usually ebbs even to a disbelief in drug action. A small number of drugs, meeting everyday indications, should be employed in the greater part of a dentist's work. Constant acquaintance familiarizes him with their nature and their uses, and with these few remedies his best work is usually done. Therapeutic nihilism is just as erroneous as the polypharmaceutic shotgun prescriptions of our ancestors. Practitioners of large experience usually obtain the best results with a few of the simple remedies, while many of the younger disciples seize upon new compounds because they do not know how to employ either of them. "When called to guide a patient through an illness, the physician should be constantly a watchman, and a therapeutist only when necessity arises."

In the following discussion of the supposed efficacy of a few dental remedies no effort has been made to arrange them in order of their importance or according to a specific classification. They have been picked out at random as being possibly more often used than others, but the list may be readily extended.

### POTASSIUM CHLORATE NOT A SPECIFIC IN STOMATITIS.

Potassium chlorate as a remedy for the various forms of stomatitis is looked upon by many practitioners as a veritable panacea. As someone has said, "It is one of the most reliable remedies, indeed almost a specific in ulcerations of the mouth, particularly in stomatitis etc." Others have lauded it as a prophylactic in mercurial stomatitis, claiming that it will prevent salivation. Potassium chlorate has been introduced into therapeutics on the erroneous theory that it will decompose in the body and supply oxygen to the tissues. It is supposed to be largely excreted by the saliva, and thereby to continuously bathe the diseased mouth with this oxygen-laden fluid. While potassium chlorate is very readily absorbed by the gastro-intestinal tract, it does not part with its oxygen at the temperature of the tissues, in fact, it is excreted almost wholly unaltered. About ninety per cent. is removed with the urine, the remainder passing out through the salivary glands. In consequence of its resistance to decomposition in the tissues, it possesses very little, if any, value as an antiseptic, and its therapeutic effect depends probably wholly on its "salt" action. This fact decides its worthlessness as a prophylactic in mercurial stomatitis. In the mouth it acts as a ready solvent for accumulated mucus, and imparts to the mucous surfaces a cool saline taste. On the other hand, it should be remembered that potassium chlorate acts as a dangerous poison. When absorbed by the blood the greater part of the hemo-globin is either directly destroyed or changed to methemoglobin, and the typical picture of chlorate poisoning is, consequently, always accompanied by pronounced cyanosis. "Increased experience has also taught us that next to the potassium cyanid, it is one of the most poisonous of the potash salts which are used in medicine." The absorption of potassium chlorate from the grastro-intestinal canal occurs occasionally most rapidly. Sollmann reports a case in which death occured within two and one-half hours after the chlorate had been taken. While the lethal dose of potassium chlorate varies very much, the quantity of fifteen grains has been fatal in a child. In a case recently reported a girl of seventeen had swallowed, during gargling, a more or less large portion of a potassium chlorate solution; within a week's time she died of typical chlorate poisoning. Quite a number of similar cases may be readily cited from the literature. Potassium chlorate in tablet form for throat trouble is in common usage by the laity; such practice should be condemned. Some years ago, Unna of Hamberg advocated the use of a tooth paste containing fifty per cent. of potassium chlorate for the treatment of mercurial stomatitis. Since then this paste has been widely advertised in the United States and Europe as being a veritable panacea in all forms of mouth diseases, including of course the *bete noire* of all oral disturbances, pyorrhea alveolaris. Lured by the attrative advertisements some dentists have made it a practice to prescribe this paste to their *clientele* for daily use. Aside from the disagreeable salty taste, the continued use of this paste is very prone to produce inflamed and easily bleeding gum margins, which, however, usually promptly disappear on discontinuing the paste and substituting a warm one per cent. sodium chlorid solution. Potassium chlorate should be eliminated from the medicinal armamentarium of the dental practitioner.

### TANNIC ACID, GALLIC ACID, ADRENALIN, AND SUGAR OF LEAD NOT INTERNAL HEMOSTATICS.

The treatment of local dental hemorrhage by the internal administration of drugs is still frequently discussed in current literature. Such remedies as tannic or gallic acid adrenalin chlorid solution, and sugar of led, with or without opium are often referred to as possessing some mystic influence in regard to the checking of hemorrhage. In most cases this conception is based upon false analogy. While the drugs here enumerated possess definite value as styptics in the milder forms of hemorrhage when locally applied, they are valueless when given internally with the hope of acting through the blood. Tannic acid is changed in the intestinal canal into gallic acid, and the very largest part of it is absorbed as sodium gallate, and as such it possesses no styptic action whatsoever. Gallic acid is practically valueless as a medicinal compound, and it may, without entailing any loss, just as well be eliminated entirely from therapeutics.

Regarding the internal administration of adrenalin or its many synomymous compounds, it should be remembered that it is completely destroyed after it is absorbed from the intestines and before it reaches the blood. This is also true when it is injected into thick muscle tissue. On the other hand, when it is injected into the circulation, even in minute doses, it causes a quick and powerful rise of blood pressure. If it is injected into a vein with the hope of constricting the vessel wall and thereby permitting the formation of a clot, it should be remembered that the great rise in blood pressure will increase the hemorrhage, and consequently its use for such purposes may be dangerous. Locally applied upon mucous surfaces or injected into the tissues, adrenalin produces definite anemia within the affected area by acting on the peripheral vessels, and for this reason it is sometimes employed as a useful styptic in minor hemorrhage. Lead acetate in small doses, usually combined with opium, is often spoken of as being a panacea in the treatment of dental hemorrhage. There is no reason justifying such a conception. Lead acetate acts as an astringent and a styptic only when applied locally upon mucous surfaces. The prolonged internal administration of lead salts is always dangerous, as it is prone to result in lead poisoning.

### SULFURATED LIME, POTASSIUM IODID, AND SARSAPARILLA AS ABORTIVES IN SUPPURATION.

Sulfurated lime, with or without iodin or potassium iodid, either alone or combined with syrup of sarsaparilla, is accredited with possessing special power for preventing or arresting suppuration. For this reason it is often advocated as an abortive in acute alveolar abscesses or other forms of suppuration about the jaws. The effect of sulfurated lime, when given internally, depends solely upon the small quantities of hydrogen sulfied which it gives up in the intestines it acts there as a local irritant. No remedial influence upon the circulation is observed from its internal administration, except that, when continually used, it acts as a dangerous poison. Potassium iodid is a most valuable drug, and under suitable conditions it may be spoken of as being a specific—as, for instance, in the treatment of sclerosis and other sequences of syphilis. It is also accredited with being a valuable adjunct in the elimination of metallic deposits from the tissues, especially in chronic metal poisoning—but right at this point its known action ceases. All the other wondrous cures which are ascribed to its action are pure and simple sophistries. Among other diseases, it has been recommended as an abortive in acute dento-alveolar abscesses, but no tangible proof of this assertion has so far been demonstrated.

Of the vegetable drugs, sassafras, guaiac, sarsaparilla, etc., share an old reputation as being highly efficacious alteratives; the latter has been much lauded as being especially efficacious, and is still widely used in the treatment of syphilis. This belief is wholly unfounded; the empiric use of sarsaparilla in the form of a syrup, decoction, etc., as a vehicle for potassium iodid or other similar compounds has no influence on this disease, an alveolar abscess, or any other disease. Sarsaparilla, senega, and other drugs of this category contain as their principal constituent the glucosid saponin, which, while soluble in water, is *not* absorbed from the grastro-intestinal canal.

### MORPHINE AND ARSENIC FOR DESTROYING THE PULP.

In 1836 Shearjashub Spooner recommended to the dental profession the use of arsenic for the purpose of destroying the pulp. "Arsenic is the only substance with which we are acquainted that will effectually destroy the pulp of a tooth. . . . Of late, we have applied to aching teeth a mixture composed of arsenic three parts and acetate of morphin one part, the morphin—one of the active principles of opium—being the most powerful odontologic remedy which we know of." Later he added creasote as a vehicle to the mixture. This combination has been used for the above purpose the world over. Innumerable other combinations have been suggested, the prime object having always been to incorporate with the arsenic some powerful local anesthetic for the purpose of mitigating the more or less severe pain following the application of the arsenic. Morphin when applied locally, has no anesthetic or narcotic effort on sensory nerve endings, and consequently it acts merely as a diluent of the arsenic. Cocain in the form of the alkaloid or its various salts and its many substitutes have been suggested for the same purpose. Scientific proof of this supposition has certainly never been brought forward In a future publication we expect to substantiate this claim more fully. A more rational procedure consists in applying to an aching pulp a concentrated solution of a local anesthetic—cocain, novocain, etc.—*prior* to the introduction of the arsenical paste.

### ALCOHOL AND SODIUM OR MAGNESIUM SULFATE AS ANTIDOTES IN PHENOL POISONING.

Alcohol, sodium sulfate—Glauber's salt and magnesium sulfate—Epsom salt—are credited with possessing definite antidotal properties in the treatment of poisoning with phenol—carbolic acid. Alcohol possesses a greater solvent power on phenol than water, hence lavage of the stomach with a ten per cent. alcohol solution in phenol poisoning is of value in the very early stages, *i. e. before* the poison is absorbed. Locally applied on a fresh phenol burn, alcohol is, for the same reason, of much benefit. It should be remembered, however, that its action is solely mechanical. As is well known, phenol combines readily with sulfuric acid in the tissues and forms a comparatively harmless body. From teleologic reasoning, readily soluble sulfates, such as magnesium or sodium sulfate, have been recommended as antidotes in phenol poisoning, with a hope of forming some non-poisonous substances. This reaction does not occur in the tissues, as Brown and Sollmann have clearly demonstrated (1906), and consequently their administration for the above purposes is totally useless.

### LITHIUM SALTS AND THE URIC ACID SOLVENTS IN GOUTY PYORRHEA ALVEOLARIS.

Things clothed in mystery give rise to much unsound speculation. This is equally true of subjects pertaining to medicine or dentistry. A significant example of this truism may be cited as an illustration by referring to the treatment of gouty pericementitis with lithium salts. Usually, we understand that—"Gout is characterized by the deposition of mono-sodium urate crystals in various parts of the body, especially in the hyalin and fibrous cartilages, in the tendons, in the subcutaneous and intramuscular connective tissues, and in the kidneys." If uric acid is present in the blood and in the urine of a patient afflicted with pyorrhea, it should be demonstrated by a careful urine analysis before the patient is subject to medical treatment. There is probably no other field in therapeutics about which so little truth is known and about which so much poetry is written as in the treatment of gout. Apparently, however, this is not true in the mind of the nostrum-maker. To him the bugbear of so-called uric acid diathesis has been and still is the very shibboleth of uncounted possibilities. Since, practically, we know nothing of the theory of gout, except that it is associated in some way with a perversion of the uric acid metabolism, our methods of treatment are necessarily futile. An increased excretion of uric acid is favored by many clinicians, and here, at least, fairly good results may be obtained by materially increasing the urine secretion. The simplest means for accomplishing this purpose is the copious drinking of water—ordinary table water or mild alkaline mineral waters will answer equally well. It is claimed that certain alkaline metallic salts, especially the various salts of lithium, possess a definite solvent power on uric acid This false deduction is based on the fact that lithium urate is about four times as soluble as sodium urate. However, the quantity of lithium carbonate which can be administered with safety is much too small to produce any effect on the blood, and lithium salicylate and benzoate, being neutral salts, do not alter the blood at all. Nevertheless, lithium salts are widely used and their value may be classed as belonging chiefly to suggestive therapeutics. A patient may forget or even object to carrying out the instruction in regard to the drinking of large quantities of water, which, in his estimation, may be of little consequence, while, on the other hand, a prescription calling for lithium carbonate or citrate tablets, with the proper directions, may readily overcome this difficulty.—*Cosmos*, Dec., 1911.

